# CRISPR/Cas9 for Cancer Therapy: Hopes and Challenges

**DOI:** 10.3390/biomedicines6040105

**Published:** 2018-11-12

**Authors:** Marta Martinez-Lage, Pilar Puig-Serra, Pablo Menendez, Raul Torres-Ruiz, Sandra Rodriguez-Perales

**Affiliations:** 1 Molecular Cytogenetics Group, Human Cancer Genetics Program, Centro Nacional de Investigaciones Oncológicas (CNIO), 28029 Madrid, Spain; mmlage@cnio.es (M.M.-L.); ppuig@cnio.es (P.P.-S.); 2 Josep Carreras Leukemia Research Institute and Department of Biomedicine, School of Medicine, University of Barcelona, 08036 Barcelona, Spain; pmenendez@carrerasresearch.org; 3 Instituciò Catalana de Recerca i Estudis Avançats (ICREA), 08010 Barcelona, Spain; 4 Centro de Investigación Biomédica en Red de Cáncer (CIBER-ONC), ISCIII, 08010 Barcelona, Spain

**Keywords:** CRISPR, cancer models, genome engineering, Cas9, advance therapy

## Abstract

Cancer is the second leading cause of death globally and remains a major economic and social burden. Although our understanding of cancer at the molecular level continues to improve, more effort is needed to develop new therapeutic tools and approaches exploiting these advances. Because of its high efficiency and accuracy, the CRISPR-Cas9 genome editing technique has recently emerged as a potentially powerful tool in the arsenal of cancer therapy. Among its many applications, CRISPR-Cas9 has shown an unprecedented clinical potential to discover novel targets for cancer therapy and to dissect chemical-genetic interactions, providing insight into how tumours respond to drug treatment. Moreover, CRISPR-Cas9 can be employed to rapidly engineer immune cells and oncolytic viruses for cancer immunotherapeutic applications. Perhaps more importantly, the ability of CRISPR-Cas9 to accurately edit genes, not only in cell culture models and model organisms but also in humans, allows its use in therapeutic explorations. In this review, we discuss important considerations for the use of CRISPR/Cas9 in therapeutic settings and major challenges that will need to be addressed prior to its clinical translation for a complex and polygenic disease such as cancer.

## 1. Mechanism and Advantages of CRISPR Genome Editing

The discovery of clustered regularly interspaced short palindromic repeats (CRISPR) [1,2,3] and their function together with CRISPR-associated (Cas) genes as an adaptive prokaryotic immune system [4,5,6,7] has paved the way for their adoption as a powerful genome-engineering tool [8,9,10,11]. Because of its specificity, efficacy, and simplicity, the CRISPR/Cas9 system has been called the biggest biotechnological discovery of the century, and has opened new possibilities for precise genome editing and in vivo imaging. Overall, CRISPR/Cas9 has shown an unprecedented clinical potential to study and target disease and to offer new avenues for drug discovery. Perhaps more importantly, it brings the promise of new diagnostic and therapeutic interventions.

**Figure 1:**
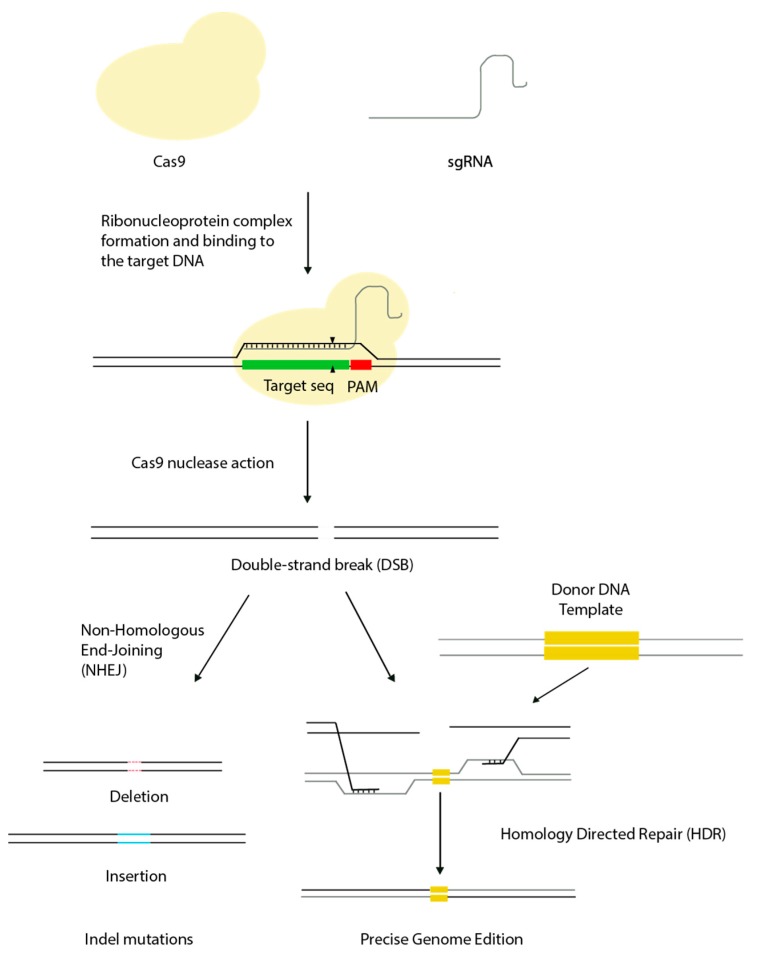
Clustered regularly interspaced short palindromic repeats (CRISPR)/Cas9 mechanism of action. Protein Cas9 binds to the sgRNA, forming a ribonucleoprotein complex (RNP). This complex then anneals to the genomic target sequence with base-pairing complementarity and specifically cleaves double-stranded DNA (black triangles) after the recognition of the protospacer-adjacent motif (PAM) sequence adjacent to the target sequence. Double-strand breaks generated activate non-homologous end joining (NHEJ) or homology-directed repair (HDR) pathways. In the absence of a homologous repair template NHEJ usually result in indels (insertions or deletions) of random base pairs disrupting the target sequence. Alternatively, precise genome edition can be made by providing a donor DNA template and exploiting the homology directed repair pathway.

Gene editing technologies are based on the generation of double-strand breaks (DSBs) in defined regions of the genome and their subsequent repair by cellular processes. In contrast to earlier approaches for genome editing, such as zinc-finger nucleases (ZFNs) and transcription activator-effector nucleases (TALENs), the RNA-guided DNA targeting CRISPR/Cas platform was quickly and widely adopted by researchers by virtue of its affordability, scalability, and ease of use, and has revolutionized the field of genome engineering. Clustered regularly interspaced short palindromic repeats/Cas9 technology has its origin in an immune defence mechanism found in bacteria and archaea, which provides immunity to the host against invading nucleic acids such as viruses and phages [12]. According to the most popular classification, there are three types of CRISPR/Cas systems, each with several subgroups [13]. The most commonly used system for gene editing is the type II CRISPR/Cas system, which consists of three components: an endonuclease (Cas9), a CRISPR RNA (crRNA), and a transactivating crRNA (tracrRNA) [8]. The crRNA and tracrRNA molecules form a duplex structure called the guide RNA (gRNA) that can be replaced by a synthetic fused chimeric single gRNA (sgRNA), which simplifies the use of CRISPR/Cas9 in genome engineering [8]. The sgRNA contains a unique 20 base-pair (bp) sequence that is designed to be complementary to the target DNA site, and this must be followed by a short DNA sequence termed the ‘‘protospacer-adjacent motif’’ (PAM), which is essential for compatibility with the Cas9 protein used. Once the sgRNA and the Cas9 nuclease are expressed in the cell, they form a ribonucleoprotein (RNP) complex that will be guided by the sgRNA to a target DNA site. The sgRNA binds to the target sequence by Watson-Crick base-pairing and Cas9 precisely cleaves the DNA to produce a DSB. The cleavage occurs within the protospacer, specifically three nucleotides upstream the PAM, generating blunt ends. Both RuvC and HNH active-site motifs of Cas9 are responsible for the cleavage of opposite DNA strands, they act on the (−) and (+) strands, respectively [14,15]. The cell machinery then repairs this DSB by one of two main mechanisms—homology-directed repair (HDR) and non-homologous end joining (NHEJ) [16,17,18]—depending on the cell state and the presence of a repair template. The HDR pathway uses a donor DNA template that is recombined at the DSB site, resulting in accurate repair. Homology-directed repair can be exploited to introduce specific sequences or mutations into a target region of the genome. The more prevalent NHEJ pathway is an error-prone system that randomly inserts or deletes nucleotides at the DSB site (indels) and can therefore be used to induce specific gene knockouts (KO) through the generation of frameshift mutations (Figure 1).

Clustered regularly interspaced short palindromic repeats/Cas9 technology thus allows for precise and highly efficient cleavage of a desired target DNA sequence, and given the relative ease and simplicity of designing sgRNAs, it has significantly facilitated genome editing. An added advantage of this technology is its potential for multiplexibility through the use of different sgRNAs. Among nucleases for genome editing, only the CRISPR/Cas9 system can edit multiple loci simultaneously by the introduction of sgRNAs targeting different sites [19,20,21]. Indeed, using two sgRNAs in the same cell can lead to the generation of small deletions [10], complex rearrangements [22,23], and even whole chromosome suppression [24]. Another important advantage of CRISPR/Cas9 is its flexibility: modifications and customizations of CRISPR/Cas9 components and interactors have not only improved the specificity and efficiency of the system but have also extended the scope of its applications beyond editing [25].

Improving the DNA specificity of CRISPR technology has been a major priority in the field, as several studies have demonstrated the presence of off-target activity [26]. Accordingly, several strategies have been developed to decrease off-target products. One such strategy is to exchange plasmid delivery for cellular delivery of in vitro-assembled RNP complexes, which results in longer-lasting Cas9 and sgRNA expression, in addition to increasing the ratio of on-target:off-target genome editing in mammalian cells, and producing highly efficient editing [27,28,29,30,31]. Other strategies include the use of Cas9 variants engineered to be inducible by light or small molecules, [32,33,34] or split Cas9 variants (for controlled reassembly) and allosterically-regulated Cas9 [35,36,37]. Modification of Cas9 to induces nicks in just one strand of the DNA have allowed the use of two Cas9 nickases, guided by two different gRNAs targeting the same locus but at opposite DNA strands. This strategy results in very specific DNA cleavage, reaching efficiencies comparable with normal CRISPR/Cas9 but limiting off target events. [38]. A similar strategy is the use of two catalytically inactive Cas9 mutants (guided by two different gRNAs targeting the same locus) fused to FokI nuclease (fCas9), such that the FokI nuclease is only functional when dimerized. fCas9 nucleases were shown to modify a target locus with >140-fold higher specificity than wild-type Cas9 nuclease in human cells [39]. Finally, mutational analysis of Cas9 to increase its specificity has shown that 3–4 engineered point mutations are able to neutralize nonspecific electrostatic interactions between Cas9 and its target DNA, significantly increasing the specificity of its action [40,41].

While there are many advantages of CRISPR/Cas9 technology over previous genome editing programmable nucleases, some limitations remain. As a relatively new technology, the efficiency and sequence specificity of CRISPR/Cas9 needs to be further improved. Also, off-target effects have to be reduced and the development of an effective, safe and cell-specific CRISPR/Cas9 delivery system remains a major challenge.

## 2. CRISPR in Drug Discovery

Drug discovery and development is a long and complex process of identifying new drugs and bringing them to market. This process typically begins with the hypothesis that perturbing a particular biological target will produce a beneficial effect that changes the course of a disease. These targets must be validated in physiologically relevant preclinical animal models whose pharmacological modulation may yield the desired therapeutic effect.

In the field of oncology, drug discovery endeavors to identify molecules against genetic aberrations in oncogenes and tumour suppressor genes that lead to tumour development. Some successful examples include imatinib, which targets *BCR–ABL*1 fusions in chronic myeloid leukaemia; vemurafenib, which targets *BRAF* V600E mutations in melanoma; or osimertinib for the treatment of EGFR-mutated non-small cell lung cancer [42].

Genome engineering is particularly useful in drug discovery programs to identify genes that are responsible for a particular disease. However, this is commonly a laborious and time-consuming process. The implementation of the CRISPR/Cas9 system, however, has the potential to accelerate the identification and validation of high-value targets. Indeed, the rapid and efficient generation of precision disease models, cellular or whole animal, by CRISPR/Cas9 engineering, should have a positive impact on drug discovery as a faster route for functional drug screening through the identification of target molecules whose activation or inhibition causes or prevents disease [43].

## 3. CRISPR/Cas9 Library Screens for Drug Target Discovery

The identification of unknown genes and determination of their function is commonly carried out with high-throughput genetic screening platforms. Mutagenesis screenings have been successfully used to discover many basic biological mechanisms and signalling pathways, and through this approach, one can determine which genes are responsible for a given phenotype. The main limitation with mutagenesis screenings for target drug discovery, however, is the generation of heterozygous mutants with unknown random mutations. One way to overcome this limitation is the use of targeted RNA interference (RNAi). High-throughput RNAi genomic library screens have provided important information on causal links between individual genes and loss-of-function phenotypes, although some limitations still remain, such as inefficient knockdown (partial knockdown) and major off-target effects. [43]. Along this line, the use of CRISPR/Cas9 provides some advantages over RNAi including complete inactivation (total knockdown), high reproducibility, and the capability to target the whole genome, including enhancers, promoters, introns, and intergenic regions [44]. The knowledge gained from the construction of functional RNAi platforms has allowed the rapid development of CRISPR/Cas9 libraries in recent years. These CRISPR libraries were first reported in 2013 to be more efficient that RNAi libraries [44,45].

**Figure 2:**
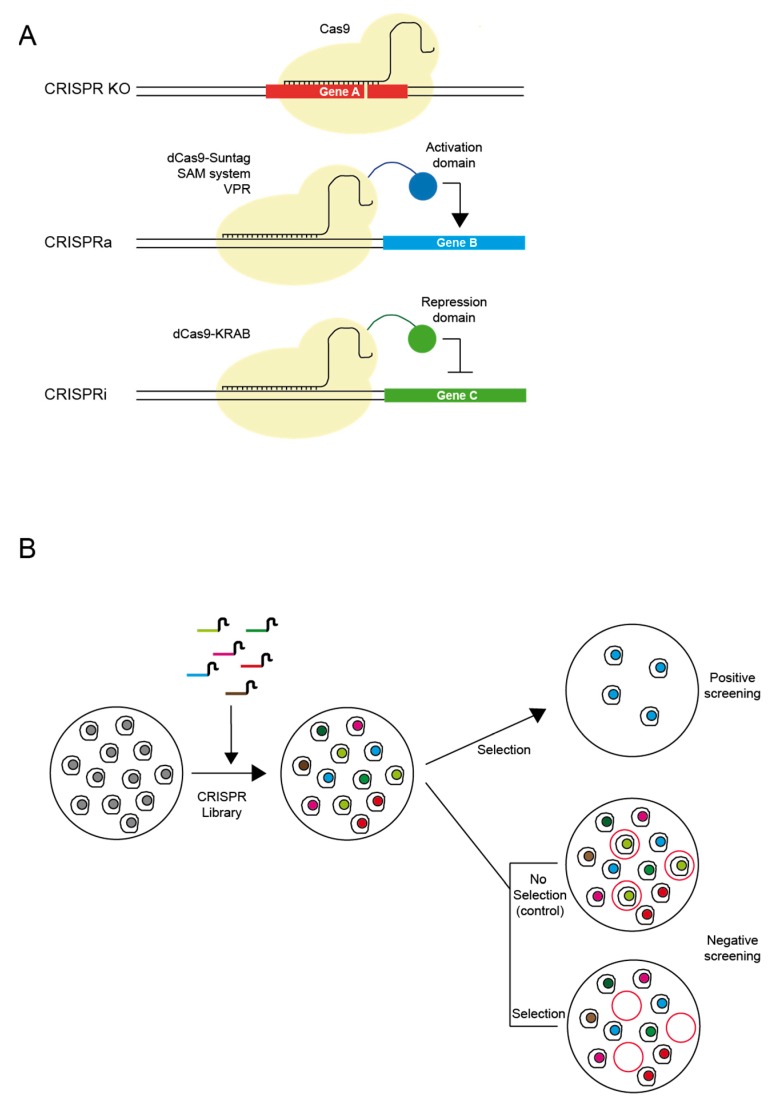
Principal applications of CRISPR used in drug discovery. (**A**) Three main approaches for transcriptional modulation: (i) CRISPR KO, to generate non-functional proteins or knock-outs of a specific gene by using wild-type CRISPR system; (ii) CRISPR activation, to produce specific gene activation using a catalytically-inactive version of the Cas9 enzyme (dCas9) fused to different activator domains (e.g., SunTag, SAM, VPR); (iii) CRISPR repression or inactivation, by fusing repressor domains (e.g., KRAB) to the dCas9 protein. (**B**) Pooled high-throughput screening can be performed using genome-scale guide RNA libraries, either in a positive or negative selection manner.

Three different types of genome-wide CRISPR libraries are currently used: (1) CRISPR-based loss-of-function (CRISPR knock-out) is used to identify new biological mechanisms including drug resistance and cell survival signals [45]; (2) CRISPR-based gene activation (CRISPRa), which is useful in screening for gain of function [46]; and (3) CRISPR-based gene inhibition (CRISPRi), which is used in the screening for loss of functions [47]. Whereas CRISPR knock-out libraries typically utilize unmodified Cas9, CRISPRa and CRISPRi libraries utilize catalytically deactivated Cas9 (dCas9) in concert with regulatory cofactors such as VP64 (activation) [48] or the Krüppel associated box (KRAB) repression (inhibition) [49], or other factors including VP64-p65-Rta (VPR), Synergistic Activation Mediators (SAM) or SunTag [50,51], developed to accelerate CRISPRa activity (Figure 2).

The flexible format of CRISPR makes it possible to perform positive and negative selection screenings. Positive selection screens identify genes that allow cells to survive under specific conditions such as a drug treatment. For example, cells can be treated with a CRISPR library and then exposed to an anti-cancer drug. Only drug-resistant survivors can be harvested to analyse the sequence of the gRNAs, which are used to identify candidate genes for drug resistance [52]. By contrast, negative selection is used to detect dead or slow-growing cells efficiently under a specific condition. This is useful to identify genes essential for survival, which can be promising candidates for molecularly targeted drugs. For example, if a pool of gRNAs is used to make a set of random mutants, those cells that carry gRNAs targeting a survival-essential gene will not survive, and after several passages only surviving cells with targeted non-essential genes will remain. Thus, by sequencing a pool of gRNAs from the initial status and survival status (using next-generation sequencing), it would be possible to identify those survival-essential candidate genes.

## 4. CRISPR/Cas9 in Drug Resistance

An important application of CRISPR/Cas9 in drug discovery is the identification of genes involved in drug resistance. Previously, the mechanisms of resistance to anticancer agents were evaluated by global mutagenesis across a cell population. Subsequent application of the drug to be tested would lead to the survival of only those cells carrying a mutation that impairs the action of the drug. The main limitation of this approach, however, is the significant number of false positives generated [53].

Clustered regularly interspaced short palindromic repeats/Cas9 screens are particularly suitable to detect gene deletions associated with drug resistance. Accordingly, cells that acquire resistance to the drug of interest are exposed to a pool of CRISPR/Cas9 gRNAs that target various genes such that there is only one guide per cell and one gene knocked out. Genes that confer drug resistance are identified via analysis of the cells that become sensitive to the drug exposure. Those genes identified as resistant to the drug can then be targeted with other drugs to avoid the emergence of resistance [54]. For example, the disruption of the *HPRT1* gene through CRISPR/Cas9 editing generates cells’ resistant to 6-thioguannie (a conventional anti-cancer drug) [55]; similarly, the homozygous C528S mutation in the *XPO1* gene mediated by CRISPR/Cas9 editing confers resistance to selinexor [56].

## 5. Disease Models for Drug Efficacy

Cell and animal models of human disease are crucial elements for drug development. Experiments to test drug efficacy and toxicity must be performed in models prior to clinical testing in humans. However, many of the available models (including cancer cell lines and animal models) do not always mirror the combination of aberrations seen in patients. Clearly it would be prohibitively expensive and time consuming to produce models that accurately recapitulate the complexity and variety of human diseases. Nevertheless, CRISPR/Cas9 has been used extensively to modify cancer cell lines to mimic aberrations seen in patients more quickly and cheaply than standard protocols. A good example of this is an approach used in the mouse ovarian cancer model ID8, which was modified to inhibit *TP53* and *BRCA2*, resulting in a sensitivity increase to PARP inhibition [57].

These examples serve to illustrate that the CRISPR/Cas9 platform has rapidly become an essential component of the drug discovery process in oncology. This technology has accelerated the identification and validation of new drug targets, and in addition has provided more robust models of human diseases to test drug safety in a more predictive manner and to reduce or combat drug resistance.

## 6. CRISPR in Cancer Therapy

Despite some progress in the last decades, the number of people that still die as a result of cancer demonstrates the urgent need of novel and more efficient therapeutic options. In addition to being a formidable research tool, CRISPR/Cas9-mediated genome editing holds immense promise in cancer therapeutic applications. A possible application of the CRISPR/Cas9 system to cancer therapy is related to the regulation of endogenous gene expression. As mentioned above, catalytically inactive dCas9 can be recruited by gRNAs to specific target DNA sites [58], and when fused to transcriptional activation or inhibition domains, can be exploited to activate or repress specific target genes [59]. Another therapeutic application could be based on the tethering of dCas9 to histone modifiers and proteins involved in altering DNA methylation, to perform targeted “epigenome editing” [60]. Considering that many epigenetic factors are involved in multiple types of cancer, such as acute lymphoblastic leukaemia or Ewing sarcoma [61], targeting the epigenetic regulatory machinery may be an effective means to dysregulate cancer. Finally, it might be possible to directly target of tumour markers in cancer cells, offering the possibility to eliminate the genetic alterations leading to tumour proliferation and/or metastatic capacity [62]. However, a caveat to this approach is the identification of bona fide driver genetic alterations involved in cancer cell viability. Another challenge would be the effective delivery of the CRISPR components into all cancer cells.

As previously mentioned, cancer is a complex disease, and effective immunity against cancer cells involves elaborate interactions between tumour, host, and environment. During the last few years, immunotherapy has emerged as a promising option to treat cancer by enhancing the immune response to tumour cells with synthetic chimeric antigen receptor (CAR) therapy or targeting the programmed death receptor 1 (PD-1) [63]. Cancer immunotherapy has many advantages over chemotherapy or radiotherapy, including favourable benefits, low risk ratio, and durable activity. The term cancer “immunotherapy” encompasses a wide variety of methods to increase tumour immunity. The development of new generation therapy approaches is especially interesting for those types of cancer that are untreatable with standard chemo- or radiotherapy regimens.

Oncolytic viruses are emerging as important agents in cancer therapeutics. These viruses can be genetically modified to lack virulence against normal cells, but maintaining its ability to attack and lyse cancer cells with deficient antiviral defences. Direct cellular lysis is one of various mechanisms involved in the viral-induced destruction of cancer cells, which triggers further immune stimulation through tumour antigens released from the dying cell [64]. Between other research and more translational applications, CRISPR/Cas9-mediated genome editing holds immense promise in cancer therapeutic applications as it could be used to engineer oncolytic viruses for optimized tumour selectivity and enhanced immune stimulation. Some examples of genomic alteration use for immunotherapy applications include the generation of herpes simplex virus type 1 variants with strong lytic properties, engineered by deletion of the ICP34.5 neurovirulence and ICP6 (UL39) (ribonucleotide reductase) genes [65]. Another example is the deletion of ICP6 to provide replicative selectivity for cells with p16^INK4A^ tumour suppressor gene inactivation, one of the most common deficiencies in cancer [66]. In the case of the DNA tumour virus, adenovirus, the wild-type form encodes a protein (E1A) that is able to bind pRb [67,68,69], releasing the transcription factor E2F and thus arresting the cell cycle. The release of E2F also triggers an organized activation of the viral genes that ultimately leads to the generation of new virions, the lysis of the infected cell, and the spread of the new virus. Because cancer cells typically have genetic alterations in the Rb pathway, the *E1A* gene has been eliminated from oncolytic adenoviruses to prevent replication and promote safety in wild-type cells.

Adoptive cell therapy (ACT) is an immunotherapy approach that involves the isolation and in vitro expansion of tumour-specific T-cells, followed by their reintroduction into the patient. There are many forms of ACT under development, including the use of T-cells that have been engineered to effectively recognize and attack tumour cells. One approach involves the deletion of the programmed cell death-1 receptor (*PD-1*) gene in T-cells [70]. The PD-1 axis has been recognized as a pivotal immune checkpoint and the interaction between PD-1 and its ligand PD-L1 inhibits T-lymphocyte proliferation, survival, and effector functions (such as cytotoxicity and cytokine release) [71], induces apoptosis of tumour-specific T-cells [72], as well as resistance of tumour cells to cytolytic T-lymphocyte attack [73,74]. The approach is based on CRISPR/Cas9-mediated *PD-1* gene deletion in T-cells ex vivo and their reintroduction into patients, where the gene-deleted T-cell will home to the tumour and activate the immune response with the possibility of tumour eradication. Immune checkpoint blockade, including the use of gene deletion or anti-PD-1/PD-L1 and anti-CTLA-4 antibodies, has led to a breakthrough in the treatment of multiple types of advanced solid tumours by preventing checkpoint molecule triggered exhaustion, and represents a powerful tool for anti-tumour treatments. Indeed, this promising approach is being tested in six clinical trials with PD-1 knockout T-cells for lymphoma, gastric, lung, prostate, and bladder cancer, and renal cell carcinoma [75] (Table S1, https://clinicaltrials.gov/).

Another exciting anticancer immune therapy that holds great promise in the treatment of haematological and solid cancers is based on the production of next-generation CAR T-cells [76], which are engineered to express tumour-targeting receptors. Chimeric antigen receptors include an intracellular chimeric signalling domain capable of activating T-cells and an extracellular binding domain that recognizes an antigen highly specific for and strongly expressed on tumour cells, working in concert to reprogram T-cell-mediated killing of tumour cells. Until recently, CAR T-cell therapy targeting the CD19 antigen has been the most studied and successful due to its specific expression in B cells and B cell leukaemia. In 2016, a team led by oncologist Lu You at Sichuan University, China, were the first to inject a patient with aggressive lung cancer with T-cells edited by CRISPR/Cas9 to disable PD-1 [77]. Two clinical trials evaluating the feasibility and safety of CD19, CD20 or CD22 CAR T-cell immunotherapy for relapse or refractory leukaemia and lymphoma have been initiated this year in China.

Although ACT therapies have shown promising results in clinical trials on leukaemia and lymphoma, some patients have died during the trial phases because of cytokine release syndrome and neurotoxicity [78]. For the moment, CAR T-cell therapy has only received approval from the FDA for the treatment of relapsed and refractory B-cell acute lymphoblastic leukaemia in paediatric and young adults [79].

## 7. In Vivo Delivery Technologies for Gene Editing

A challenge for the future application of gene editing tools, such as the CRISPR/Cas9 system, will be the development of efficient and safe methods to deliver gene-editing elements not only to the primary tumour cells, but also at the metastatic sites. To date, therapeutic ex vivo gene editing has been performed mainly in haematopoietic precursors or T-cells. To broaden the application of CRISPR-based therapy, developing efficient methods for in vivo delivery in somatic cells is indispensable. These additional delivery hurdles may be overcome with the implementation or development of new viral and non-viral systems [80,81,82,83].

Viral delivery systems for CRISPR/Cas9 components could include adeno-associated virus (AAV), lentivirus, and adenovirus [84]. Among these, AAVs are currently the most advanced methodology for in vivo gene delivery [82]. Indeed, AAV is an excellent vehicle for gene therapy for many reasons: (i) AAV is not known to cause any diseases in humans; (ii) there is a wide range of known serotypes for infection of different cell types; (iii) AAV provokes little or no immune response [85]. Moreover, AAVs have been successfully used in mouse models [86,87], and their efficacy and safety have been tested in clinical trials with recent approval [88]. One of the major drawbacks for their use, however, is their small packaging size, making it necessary to use several viruses to deliver all of the CRISPR/Cas9 components (Cas9, sgRNAs, and if necessary donor DNA), which further decreases the editing efficiency [84]. Unlike some other methods, the use of AAV provides a persistent expression of CRISPR components in edited cells, which could increase the potential immune responses or undesirable off-target genome effects. Similar to AAV, lentivirus and adenovirus can infect both dividing and non-dividing cells; however, unlike lentiviruses (and AAV at a low frequency), adenoviruses do not integrate into the genome of the recipient cell. Also, lentivirus tropism can be altered with other viral proteins, such as the G-protein of vesicular stomatitis virus (VSVG). Nevertheless, there are some drawbacks to the use of lentivirus and adenovirus, as both systems elicit strong immune responses [89,90]. All that being said, all of these viruses are highly versatile particles because they can be applied in vitro, ex vivo or in vivo, which eases both efficacy and safety testing.

In contrast to viral delivery, non-viral delivery of vectors or short-lived and preassembled Cas9 RNP complexes may provide an alternative strategy to meet these challenges. Non-viral methods include lipid nanoparticles/liposomes, gold nanoparticles or inorganic nanoparticles, among many others [81]. Lipid nanoparticles have long been used as delivery tools for a wide range of different molecules to cells. As they do not contain any viral components, they may alleviate safety and immunogenicity concerns. They can also be used in vitro, ex vivo, and in vivo. Another advantage of nanoparticle-based delivery of CRISPR elements is its high loading capacity, without the risk of genomic integration and effects from persistent expression of CRISPR/Cas9 [91]. Nanoparticle-mediated delivery of Cas9-sgRNA RNP complexes has been reported in the U2OS human osteosarcoma cell xenografts [92]. Moreover, a new vehicle composed of gold nanoparticles conjugated to DNA and complexed with cationic endosomal disruptive polymers was shown to deliver Cas9 RNP complexes plus donor DNA and could induce HDR to correct the DNA mutation of Duchenne muscular dystrophy in mice [93]. Gold nanoparticles provide non-toxic carriers for drug and gene delivery applications, in which the gold core imparts stability to the assembly, while the monolayer allows tuning of surface properties such as charge and hydrophobicity. While this method also requires additional testing, it is a promising delivery mechanism for CRISPR components. Indeed, inorganic nanoparticles are natural potential CRISPR component carriers as they have already been used for similar purposes [94], including carbon nanotubes, bare mesoporous or dense silica nanoparticles. Moreover, inorganic nanoparticles are simpler to generate, with reproducible composition, size, and stability over time.

## 8. Concluding Remarks

With its potential already demonstrated in research, CRISPR-mediated genome editing holds immense therapeutic promise; however, the successful clinical implementation of this technology will require its safe and effective delivery into target tissues. The great expectations surrounding CRISPR gene editing needs to be coupled with strategic planning, including enabling regulatory processes to ensure the successful development of this advanced gene editing-based modality. What is clear, nevertheless, is that the technology still requires optimization before widespread translation into the clinic, especially with regards to efficacy, safety, and specificity. Although some challenges remain, we envision that the continuous advancement of this gene editing technology will contribute to the improvement of current cancer treatments.

## Supplementary Materials

The following are available online at http://www.mdpi.com/2227-9059/6/4/105/s1.
